# Supplementary figures and images for: Public perspectives on COVID-19 triage protocols for access to critical care in extreme pandemic context
Source: PLoS One. 2024 Dec 17;19(12):e0314460. doi: 10.1371/journal.pone.0314460 (PMC11651564; doi:10.1371/journal.pone.0314460)

**S1 Table**

**Intercoder reliability**


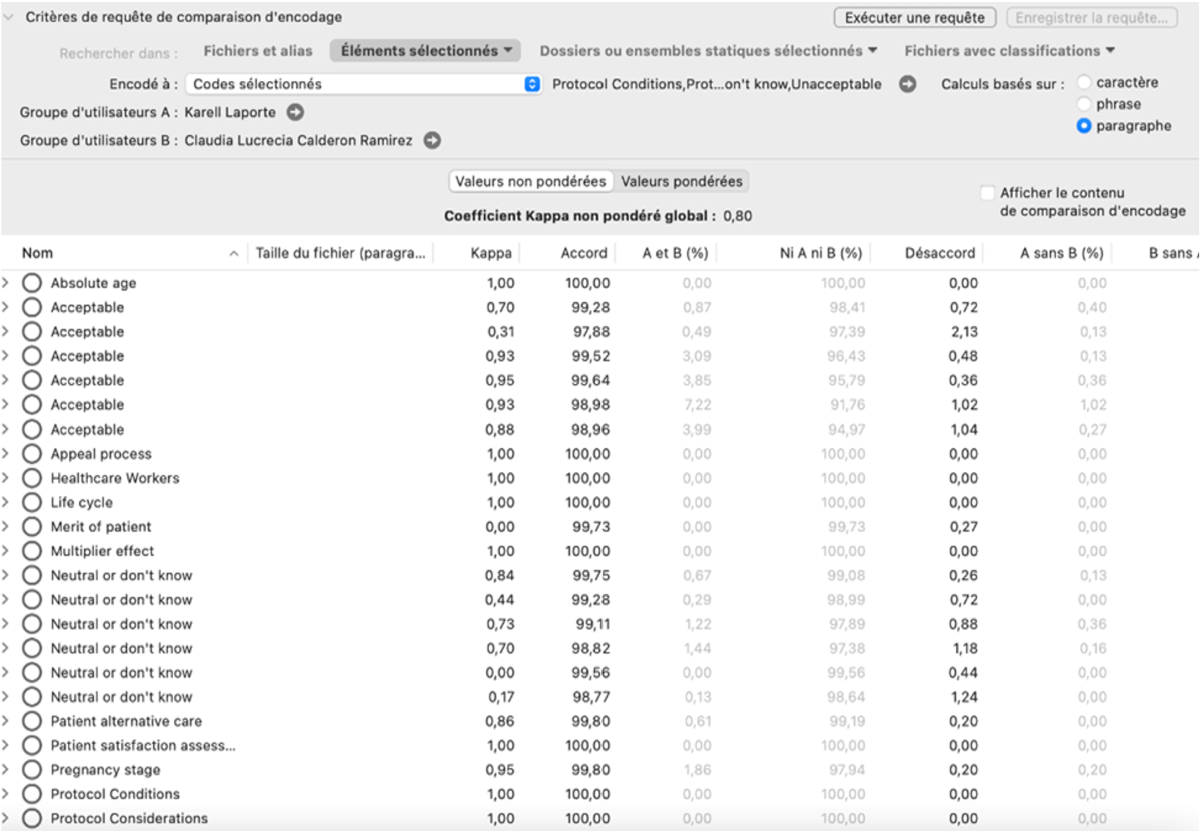

Supplement: S1 Table — (DOCX) [file pone.0314460.s001.docx]
